# Supplementary material for: The effect of mHealth on childhood vaccination in Africa: A systematic review and meta-analysis
Source: PLoS One. 2024 Feb 21;19(2):e0294442. doi: 10.1371/journal.pone.0294442 (PMC10880990; doi:10.1371/journal.pone.0294442)
Supplement: S1 File — (DOCX) [file pone.0294442.s002.docx]

**1. PubMed**

#1“childhood immunization”[title/abstract] OR # 2 “childhood vaccination”[title/abstract] OR #3”child vaccination”[title/abstract] OR #4”child immunization”[title/abstract] OR #5”rate of immunization”[title/abstract] OR #6 ” immunization coverage”[title/abstract] OR #7”immunization timeliness”[title/abstract] OR #8 “immunization completion”[title/abstract] OR #9”rate of vaccination”[title/abstract] OR #10” vaccination coverage”[title/abstract] OR #11”vaccination timeliness”[title/abstract] OR #12“vaccination completion”[title/abstract]

#13“Mobile Applications”[MESH]

#14“Telemedicine”[MESH]

#15“Text Messaging”[MESH]

#16“Cell Phone”[MESH]

#17“Smartphone”[MESH]

#18“Mobile Applications” OR “mobile” OR “Telemedicine” OR “telephone” OR “Text Messaging” OR "Telenursing" OR “Cell Phone” “cellular phone” OR “phone” OR tablet* OR ipad* OR ipod* OR android OR nexus OR iOS OR handheld* OR hand-held* OR notebook* OR "note book*" OR “multimedia message” OR “multi-media message” OR “Smartphone” OR “smart-phone” OR “mHealth” OR “Mobile” OR “Portable Software Application” OR “Tele*” OR “m-Health” OR “?phone*” OR “Text*” OR “Short Message” OR “SMS” OR “App” OR “Apps” OR “App-based” OR “Electronic” OR “Message*” OR “Web” OR “Web-based” OR “Internet*” OR “Digital*”

#19(randomized controlled trial[pt] OR controlled clinical trial[pt] OR randomized[tiab] OR placebo[tiab] OR clinical trials as topic[mesh:noexp] OR randomly[tiab] OR trial[ti]) NOT (animals [mh] NOT (humans [mh] AND animals[mh]))

#20([Algeria](http://listofafricancountries.com/Algeria) OR [Angola](http://listofafricancountries.com/Angola) OR [Benin](http://listofafricancountries.com/Benin) OR [Botswana](http://listofafricancountries.com/Botswana) OR [Burkina Faso](http://listofafricancountries.com/Burkina+Faso) OR [Burundi](http://listofafricancountries.com/Burundi) OR [Cameroon](http://listofafricancountries.com/Cameroon) OR [Canary Islands](http://listofafricancountries.com/Canary+Islands) OR [Cape Verde](http://listofafricancountries.com/Cape+Verde) OR [Central African Republic](http://listofafricancountries.com/Central+African+Republic) OR [Ceuta](http://listofafricancountries.com/Ceuta) OR [Chad](http://listofafricancountries.com/Chad) OR [Comoros](http://listofafricancountries.com/Comoros) OR [Côte d'Ivoire](http://listofafricancountries.com/C%C3%B4te+d%27Ivoire) OR [Democratic Republic of the Congo](http://listofafricancountries.com/Democratic+Republic+of+the+Congo) OR [Djibouti](http://listofafricancountries.com/Djibouti) OR [Egypt](http://listofafricancountries.com/Egypt) OR [Equatorial Guinea](http://listofafricancountries.com/Equatorial+Guinea) OR [Eritrea](http://listofafricancountries.com/Eritrea) OR [Ethiopia](http://listofafricancountries.com/Ethiopia) OR [Gabon](http://listofafricancountries.com/Gabon) OR [Gambia](http://listofafricancountries.com/Gambia) OR [Ghana](http://listofafricancountries.com/Ghana) OR [Guinea](http://listofafricancountries.com/Guinea) OR [Guinea-Bissau](http://listofafricancountries.com/Guinea-Bissau) OR [Kenya](http://listofafricancountries.com/Kenya) OR [Lesotho](http://listofafricancountries.com/Lesotho) OR [Liberia](http://listofafricancountries.com/Liberia) OR [Libya](http://listofafricancountries.com/Libya) OR [Madagascar](http://listofafricancountries.com/Madagascar) OR [Madeira](http://listofafricancountries.com/Madeira) OR [Malawi](http://listofafricancountries.com/Malawi) OR [Mali](http://listofafricancountries.com/Mali) OR [Mauritania](http://listofafricancountries.com/Mauritania) OR [Mauritius](http://listofafricancountries.com/Mauritius) OR [Mayotte](http://listofafricancountries.com/Mayotte) OR [Melilla](http://listofafricancountries.com/Melilla) OR [Morocco](http://listofafricancountries.com/Morocco) OR [Mozambique](http://listofafricancountries.com/Mozambique) OR [Namibia](http://listofafricancountries.com/Namibia) OR [Niger](http://listofafricancountries.com/Niger) OR [Nigeria](http://listofafricancountries.com/Nigeria) OR [Republic of the Congo](http://listofafricancountries.com/Republic+of+the+Congo) OR [Réunion](http://listofafricancountries.com/R%C3%A9union) OR [Rwanda](http://listofafricancountries.com/Rwanda) OR [Saint Helena](http://listofafricancountries.com/Saint+Helena) OR [São Tomé and Príncipe](http://listofafricancountries.com/S%C3%A3o+Tom%C3%A9+and+Pr%C3%ADncipe) OR [Senegal](http://listofafricancountries.com/Senegal) OR [Seychelles](http://listofafricancountries.com/Seychelles) OR [Sierra Leone](http://listofafricancountries.com/Sierra+Leone) OR [Somalia](http://listofafricancountries.com/Somalia) OR [South Africa](http://listofafricancountries.com/South+Africa) OR [Sudan](http://listofafricancountries.com/Sudan) OR [Swaziland](http://listofafricancountries.com/Swaziland) OR [Tanzania](http://listofafricancountries.com/Tanzania) OR [Togo](http://listofafricancountries.com/Togo) OR [Tunisia](http://listofafricancountries.com/Tunisia) OR [Uganda](http://listofafricancountries.com/Uganda) OR [Western Sahara](http://listofafricancountries.com/Western+Sahara) OR [Zambia](http://listofafricancountries.com/Zambia) OR [Zimbabwe](http://listofafricancountries.com/Zimbabwe))

#21 #1 OR #2 OR #3 OR #4 OR #5 OR #6 OR #7 OR #8 OR #9 OR #10 OR #11 OR #12

#22 #13 OR #14 OR #15 OR #16 OR #17 OR #18

#23 #20

#24 #19 AND #21 AND #22 AND #23

**2. Web of Science**

#1“childhood immunization” OR “childhood vaccination” OR ”child vaccination” OR ”child immunization” OR ”rate of immunization” OR ” immunization coverage” OR ”immunization timeliness” OR “immunization completion” OR ”rate of vaccination” OR ” vaccination coverage” OR ”vaccination timeliness” OR “vaccination completion”

#2“Mobile Applications” OR “Telemedicine” OR “Text Messaging” OR “Cell Phone” OR “Smartphone” OR “mobile” OR “Portable Software Application” OR “Tele*” OR “mHealth” OR “m-Health” OR “?phone*” OR “Text*” OR “Short Message” OR “SMS” OR “app” OR “apps” OR “app-based” OR “electronic” OR “Message*” OR “web” OR “web-based” OR “Internet*” OR “digital*”

#3 TS= clinical trial* OR TS=research design OR TS=comparative stud* OR TS=evaluation stud* OR TS=controlled trial* OR TS=follow-up stud* OR TS=prospective stud* OR TS=random* OR TS=placebo* OR TS=(single blind*) OR TS=(double blind*)

#4“childhood immunization” OR “childhood vaccination” OR ”child vaccination” OR ”child immunization” OR ”rate of immunization” OR ” immunization coverage” OR ”immunization timeliness” OR “immunization completion” OR ”rate of vaccination” OR ” vaccination coverage” OR ”vaccination timeliness” OR “vaccination completion”

#5 #1 AND #2 AND #3 AND #4

**3. Cochrane Library**

#1“childhood immunization”

#2“childhood vaccination”

#3”child vaccination”

#4”child immunization”

#5”rate of immunization”

#6” immunization coverage”

#7”immunization timeliness”

#8“immunization completion”

#9”rate of vaccination”

#10” vaccination coverage”

#11”vaccination timeliness”

#12“vaccination completion”

#14”childhood vaccination” [MESH descriptor]

#15 “Mobile Applications”[MESH descriptor]

#16 Telemedicine[MESH descriptor]

#17 “Text Messaging”[MESH descriptor]

#18 “Cell Phone”[MESH descriptor]

#19 Smartphone[MESH descriptor]

#20 mobile OR “Portable Software Application” OR Tele* OR mHealth OR eHealth OR e-health OR m-Health OR ?phone* OR Text* OR “Short Message” OR SMS OR app OR apps OR “app-based” OR electronic OR Message* OR web OR “web-based” OR Internet* OR digital*

#21 #1OR #2 OR #3 OR #4 OR #5 OR #6 OR #7 OR #8

#22 #9 #10 OR #11 OR #12 OR #13 #14 OR

#23 #15 AND #16

**4. Embase**

#1‘childhood immunization’/exp OR ‘childhood vaccination’/exp OR ‘child vaccination’/exp OR ‘child immunization’/exp OR ‘rate of immunization’/exp OR ‘immunization coverage’/exp OR ‘immunization timeliness’/exp OR ‘immunization completion’/exp OR ‘rate of vaccination’/exp OR ‘vaccination coverage’/exp OR ‘vaccination timeliness’/exp OR ‘vaccination completion’/exp

#2'mobile application'/exp OR 'telemedicine'/exp OR 'text messaging'/exp OR 'mobile phone'/exp OR 'smartphone'/exp

#3'mobile':ti,ab,kw OR 'portable software application':ti,ab,kw OR 'tele*':ti,ab,kw OR 'mhealth':ti,ab,kw OR 'm-health':ti,ab,kw OR '?phone*':ti,ab,kw OR 'text*':ti,ab,kw OR 'short message':ti,ab,kw OR 'sms':ti,ab,kw OR 'app':ti,ab,kw OR 'apps':ti,ab,kw OR 'app-based':ti,ab,kw OR 'electronic':ti,ab,kw OR 'message*':ti,ab,kw OR 'web':ti,ab,kw OR 'web-based':ti,ab,kw OR 'internet*':ti,ab,kw OR 'digital*':ti,ab,kw

#4 ('crossover procedure':de OR 'double-blind procedure':de OR 'randomized controlled trial':de) AND or  AND 'single-blind procedure':de OR (random*:de,ab,ti AND or :de,ab,ti AND factorial*:de,ab,ti) OR crossover*:de,ab,ti OR ((cross NEXT/1 over*):de,ab,ti) OR placebo*:de,ab,ti OR ((doubl* NEAR/1 blind*):de,ab,ti) OR ((singl* NEAR/1 blind*):de,ab,ti) OR assign*:de,ab,ti OR allocat*:de,ab,ti OR volunteer*:de,ab,ti AND Africa

#5 #2 OR #3

#6 #1 AND #4

#7 #5 AND #6

**5. ClinicalTrials.gov**

mHealth OR digital OR Mobile OR Smartphone OR "Cell phone" OR Techno OR "short message service" OR SMS OR Tele OR Telemedicine OR Telehealth OR E-health OR eHealth OR Remote OR Electro OR Comput OR cloud OR Software OR Application AND “childhood immunization” OR “childhood vaccination” OR ”child vaccination” OR ”child immunization” OR ”rate of immunization” OR ” immunization coverage” OR ”immunization timeliness” OR “immunization completion” OR ”rate of vaccination” OR ” vaccination coverage” OR ”vaccination timeliness” OR “vaccination completion” AND Africa

**6. Sciencedirect**

mHealth OR Mobile OR Smartphone OR "Cell phone" OR Techno OR "short message service" OR SMS OR Tele OR Telemedicine OR Telehealth OR E-health OR eHealth OR Remote OR Electro OR Comput AND “childhood immunization” OR “childhood vaccination” OR ”child vaccination” OR ”child immunization” OR ”rate of immunization” OR ” immunization coverage” OR ”immunization timeliness” OR “immunization completion” OR ”rate of vaccination” OR ” vaccination coverage” OR ”vaccination timeliness” OR “vaccination completion” AND Africa

**7. African Journals Online (AJOL)**

mHealth OR Mobile OR Smartphone OR "Cell phone" OR Techno OR "short message service" OR SMS OR Tele OR Telemedicine OR Telehealth OR E-health OR eHealth OR Remote OR Electro OR Comput AND “childhood immunization” OR “childhood vaccination” OR ”child vaccination” OR ”child immunization” OR ”rate of immunization” OR ” immunization coverage” OR ”immunization timeliness” OR “immunization completion” OR ”rate of vaccination” OR ” vaccination coverage” OR ”vaccination timeliness” OR “vaccination completion” AND Africa

**8. WHO International Clinical Trials Registry Platform (ICTRP)**

mHealth OR Mobile OR Smartphone OR "Cell phone" OR Techno OR "short message service" OR SMS OR Tele OR Telemedicine OR Telehealth OR E-health OR eHealth OR Remote OR Electro OR Comput AND “childhood immunization” OR “childhood vaccination” OR ”child vaccination” OR ”child immunization” OR ”rate of immunization” OR ” immunization coverage” OR ”immunization timeliness” OR “immunization completion” OR ”rate of vaccination” OR ” vaccination coverage” OR ”vaccination timeliness” OR “vaccination completion” AND Africa
